# From Editor’s Desk

**DOI:** 10.4103/0973-1075.76228

**Published:** 2011-01

**Authors:** Sushma Bhatnagar

**Affiliations:** Editor, IJPC Dr. BRAIRCH, AIIMS, New Delhi E-mail: sushmabhatnagar1@gmail.com

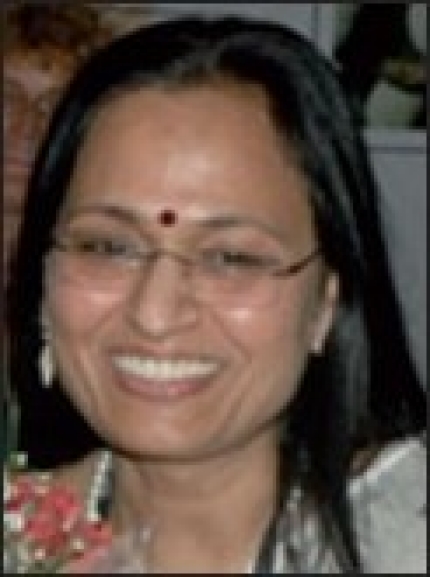



I am glad that the 18^th^ International Conference on “*Indian Association of Palliative Care*” is being organized by Cancer Aid Society at Sanjay Gandhi Institute of Medical Sciences, Lucknow, UP, from 11 to 13 February 2011. It gives me immense pleasure in writing this message through *Indian Journal of Palliative Care* on this occasion as Editor and the Scientific Chair of this conference.

The theme of the conference is “*Networking in Palliative Care*”. The main objective is to improve the knowledge base and skills in addressing the issues related to palliative care, focusing mainly on the networking aspect at this grand meet. The scientific content of this conference focuses on how to incorporate the art, science and principles of networking in palliative care. This is an opportunity for doctors, nurses and other health care professionals to have a good overview of the issues related to this subject.

This supplementary issue of *IJPC* is a collection of manuscripts including lectures to be delivered by the esteem faculties, covering almost all the aspects of palliative care and its networking. This issue also includes abstracts for oral and poster presentations provided by enthusiastic students, practicing doctors, nurses, research scientists and academicians from 10 different countries. I extend my warmest thanks to the authors for their interest, enthusiasm and timely submission of lecture write-up and participation in this mega event of more than 400 delegates.

As Editor of *IJPC*, I anticipate that this issue would be of immense value and will be definitely useful to medical science and palliative care personnel in their practice or thinking process. This collection will also offer a window for new perspectives and directions in the area of palliative care in the readers’ mind for long.

